# Transcriptome-Wide Characterization of Seed Aging in Rice: Identification of Specific Long-Lived mRNAs for Seed Longevity

**DOI:** 10.3389/fpls.2022.857390

**Published:** 2022-05-16

**Authors:** Bingqian Wang, Songyang Wang, Yuqin Tang, Lingli Jiang, Wei He, Qinlu Lin, Feng Yu, Long Wang

**Affiliations:** ^1^State Key Laboratory of Chemo/Biosensing and Chemometrics, Hunan Province Key Laboratory of Plant Functional Genomics and Developmental Regulation, College of Biology, Hunan University, Changsha, China; ^2^National Engineering Laboratory for Rice and By-Product Deep Processing, Central South University of Forestry and Technology, Changsha, China; ^3^Longping Agricultural Science and Technology Huangpu Research Institute, Guangzhou, China

**Keywords:** seed longevity, RNA-seq analysis, rice varieties, artificial aging, natural aging

## Abstract

Various long-lived mRNAs are stored in seeds, some of which are required for the initial phase of germination and are critical to seed longevity. However, the seed-specific long-lived mRNAs involved in seed longevity remain poorly understood in rice. To identify these mRNAs in seeds, we first performed aging experiment with 14 rice varieties, and categorized them as higher longevity (HL) and lower longevity (LL) rice varieties in conventional rice and hybrid rice, respectively. Second, RNA-seq analysis showed that most genes showed similar tendency of expression changes during natural and artificial aging, suggesting that the effects of these two aging methods on transcription are comparable. In addition, some differentially expressed genes (DEGs) in the HL and LL varieties differed after natural aging. Furthermore, several specific long-lived mRNAs were identified through a comparative analysis of HL and LL varieties after natural aging, and similar sequence features were also identified in the promoter of some specific long-lived mRNAs. Overall, we identified several specific long-lived mRNAs in rice, including gibberellin receptor gene *GID1*, which may be associated with seed longevity.

## Introduction

The seed is the carrier of biological genetic information and the basis of agricultural production. Seed longevity, the period over which seeds remain viable, is an important agronomical trait that determines its viability, storability, and quality (Zhao et al., 2021). Typically, seed longevity is measured using the final germination percentage and the indices of seedling percentage after aging (Zhao et al., 2021). Generally, seeds with high seed vigor germinate and emerge more quickly, are more resistant to stress and have the potential for high yield and quality in agricultural practice. Seed aging refers to the reduction in seed viability, loss of vitality and irreversible changes that result in the inability to germinate. Aging is a process that occurs along with the prolonged storage of seeds. The degree of seed aging is compounded by improper storage conditions, especially high temperature and high humidity. During the storage period, a series of harmful events will occur inside the seed, such as cell membrane damage, DNA damage and mutation, and long-lived mRNA degradation. Thus, the reduction in seed longevity is often associated with damage to nucleic acids and proteins.

Seed longevity is determined by genetic and physiological storage potential of the seeds (Qun et al., 2007; Bewley et al., 2012) and by their interaction with environmental factors and events causing deterioration during storage. Several genes controlling seed longevity in rice have been identified. For example, the transcription factor ABSCISIC ACID-INSENSITIVE3 (ABI3) plays a central role in seed longevity (Sano et al., 2016), as the *abi3* mutant is intolerant to desiccation and exhibits rapid viability loss during dry storage. Additionally, the indole-3-acetic acid (IAA)-amido synthetase gene GRETCHEN HAGEN3-2 (OsGH3-2) acts as a negative regulator of seed viability by regulating many genes related to the abscisic acid (ABA) pathway, subsequently regulating the accumulation level of ABA (Yuan et al., 2021). Gibberellin (GA) is another well-known phytohormone that control seed dormancy and germination, in a manner different from ABA. GIBBERELLIN INSENSITIVE DWARF1 (GID1) encodes a soluble GA receptor, plays important role in seed germination (Ge and Steber, 2018). The *Arabidopsis* contains 3 GID1 orthologs, named *AtGID1a*, *AtGID1b*, and *AtGID1c*, while rice contains only a single GID1 (Ueguchi-Tanaka et al., 2005; Nakajima et al., 2006). It has been shown that Gibberellic acid (GA3)-treated seeds or those of the quintuple DELLA mutant (with constitutive GA signaling) had higher artificial aging resistance, indicating that GA might play a positive role in seed longevity (Bueso et al., 2014). In addition, several QTLs controlling seed longevity in rice have been identified, and using 299 indica accessions, it was shown that eight major loci related to sugar metabolism, DNA repair and transcription, reactive oxygen species (ROS) and embryonic/root development were associated with seed longevity (Lee et al., 2019). To date, proteomic analyses revealed that changes in the regulation of posttranslational modifications, protein synthesis, and protein turnover play crucial roles in seed longevity, and that proteins associated with metabolism, energy, and protein synthesis were enriched after the artificial aging of seeds (Zhang et al., 2016).

Dry seeds accumulate various mRNAs, called long-lived mRNAs, that are thought to be translated after the onset of imbibition and to function during the early stage of imbibition (Bai et al., 2020). More than 12,000 different long-lived mRNAs have been identified in *Arabidopsis* dry seeds, and some of them are essential for seed longevity (Nakabayashi et al., 2005). Abscisic acid-responsive elements (ABREs) containing the core motif ACGT were overrepresented in the promoters of highly expressed genes in dry seeds (Nakabayashi et al., 2005). *De novo* protein synthesis during the initial phase of seed germination occurs from long-lived mRNAs stored in mature dry seeds without *de novo* transcription (Kimura and Nambara, 2010), and 17% of long-lived mRNAs that are specifically associated with monosomes are translationally upregulated during seed germination (Bai et al., 2020); thus, the translational capacity of dry seeds is important for seed vigor (Rajjou et al., 2007). High-throughput sequencing aid to identify potential seed longevity-related genes through transcriptome sequencing. For instance, several genes involved in ABA biosynthetic processes and the DNA damage response pathway has been identified through RNA-seq (Qu et al., 2020). However, more seed longevity-related genes need exploration.

Since natural aging too long, the aging process must be artificially accelerated for seed longevity research. The controlled deterioration treatment (CDT) was applied to accelerate seed aging for a short period (Rajjou and Debeaujon, 2008). It has been shown that similar molecular events accompany CDT and natural aging at the proteome level in the model plant *Arabidopsis thaliana* (Rajjou and Debeaujon, 2008). Several other aging methods, such as the artificial aging method (AA, aging at high temperature and high relative humidity) and the elevated partial pressure of oxygen (EPPO) method (Groot et al., 2012; Buijs et al., 2020), have been successfully used for seed aging study. The results of different aging methods are affected by different loci in the genome (Buijs et al., 2020; Fenollosa et al., 2020). At present, artificial aging treatment is widely used by seed companies as a vigor assay for numerous seed species to determine the mechanisms of seed vigor loss during storage (Li et al., 2017; Min et al., 2017). However, it is unknown whether natural and artificial aging are distinct on the transcriptional level.

Here, we selected 14 conventional and hybrid rice varieties and identified them as higher longevity (HL) and lower longevity (LL) varieties. RNA-seq analysis showed that most differentially expressed gene changes after naturing aging were similar to that of after artificial aging, indicating that the effects of these two aging methods on the transcription level are similar. Lastly, we identified several specific long-lived mRNAs through a comparative analysis of DEGs in HL and LL varieties after aging.

## Materials and Methods

### Seed Material and Growth Conditions

Seven conventional rice varieties and seven hybrid rice varieties were used for the follow-up experiments (Supplementary Table 1). Conventional varieties (YZX, XW13, YC, HM, YH988, XEH, NX32) were purchased from Zhangjiajie Farm (Hunan Province, China), and these seeds were planted at Changsha Observation and Research Station for Agriculture Ecosystems, Chinese Academy of Sciences (Xiangfeng Village, Jinjing Town, Changsha) under the same fertilization and management conditions, harvested in September and stored at −20°C for later analysis. The hybrid varieties LLY1353, LLYHZ, LLY1988, SLY5814, JLY1212, HR2, and LLY534 were planted in the same field and were purchased from Hunan Yahua Seed Industry Co., Ltd.

### Determination of the Initial Water Content in Rice

Prior to starting the aging tests, all seeds were dried under a constant weight with initial moisture content, the initial moisture content of the rice seeds was measured by a halogen moisture analyzer. Seeds with a moisture content higher than 15% were dried at a constant temperature of 30°C, and the water content was measured every 12 h until the moisture content was less than 15%. When the moisture content of all varieties dropped below 15% and was basically the same, drying was stopped. Seeds were then used for the aging experiment.

### Natural and Artificial Aging Treatment

The natural aging treatment was performed as follows: approximately 100 g of rice seeds of each variety that had been dried to a consistent moisture content (approximately 14%) was used, and the seeds were stored at room temperature (5–33°C) and 60–80% relative humidity in a laboratory in Changsha for 1 year.

Artificial accelerated aging treatment was performed as described by Qu et al. (2020). One hundred g seeds were wrapped in nylon bags, with 6 nylon bags for each variety, and marked as artificial aging for 0, 10, 15, 20, 25, and 30 d. The seeds in each bag were evenly placed in an artificial climate chamber (42°C, and humidity 87%) for 10–30 days.

### Germination Rate Determination and ID_50_

For each variety, approximately 10 g seeds treated with aging for different days were used for the germination experiment. The seeds were immersed in a 400-fold diluted “84” solution for 10 min and then washed with distilled water to remove floating seeds. Each sample was set three biological repetitions, 100 seeds for each repetition. These seeds were placed in a petri dish impregnated with moist filter paper. After that, the seeds were placed in an artificial climate chamber at 30°C, and water evaporation was observed every day and water was replenished if needed. After 8 days of germination, the number of germinated seeds was counted and recorded. ID_50_ refers to the time required for the seed germination rate to drop to half of the initial germination rate.

### Conductivity Measurement

The rice seeds were shelled with a small shelling machine, and 25 health rice grains were selected. After being rinsed three times with distilled water, the samples were dried with filter paper. The rice grains were placed in a 50 mL beaker, and then 20 mL of distilled water was added and soaked for 12 h at 25°C, resulting in three blank controls. Measurements were carried out using a DDS-11A digital display conductivity meter. First, the electrode was placed in distilled water for calibration before measurement, and then the conductivity values of the sample (B) and the blank control (A) were measured. The conductivity of the sample was calculated as follows: conductivity = value B − value A.

### RNA-Seq

Seeds of four varieties (LLY534, JLY1212, YZX, and NX32) that were subjected to 10 days of artificial aging or 1 year of natural aging, and those from untreated controls, were collected and immediately treated with liquid nitrogen on ice using a small-scale gluten washing machine and finally stored on dry ice. Each treatment was set two biological replicates and all samples were sent to Hangzhou Lianchuan Biotechnology Co., Ltd. for RNA-seq.

RNA-seq libraries were constructed and paired-end sequenced by Hangzhou Lianchuan Biotechnology Co., Ltd. RNA-seq analysis was performed according to Qu et al. (2020). Briefly, sequenced reads were screened, and quality-controlled sequences were mapped using HISAT2 v2.1.1 (Pertea et al., 2016). Transcript splicing and merging were conducted with StringTie 1.3.0. Normalized expression values were calculated with Ballgown. We defined genes as differentially expressed when they had a *p*< 0.05 and | log_2_FC| > 1. The sequencing data reported in this paper are summarized in Supplementary Table 2 and have been deposited in the GSA database (Genome Sequence Archive in the BIG Data Center, Chinese Academy of Sciences; PRJCA006248) (Members, 2018).

### Bioinformatics Analysis

For Gene Ontology (GO) enrichment analysis, GENEONTOLOGY^1^ was used to assess the detected DEGs according to Biological Process, Molecular Function, and Cellular Component ontologies. TBtools and Venny (version 2.1.0) were used for some gene screening work (Oliveros, 2007–2015; Chen et al., 2020), and TBtools and R software (version 3.5.1) were used for graphing.

### Motif Analysis

The sequences of rice were extracted from the Rice Genome Annotation Project,^2^ and TBtools (GXF sequences extract function) was used to extract the 5′UTR, 3′UTR and promoter sequences of each gene. DNA motif analyses were performed using the MEME suite (Bailey and Elkan, 1994), the FIMO was used for identified motif. Firstly, motif was entered in the input motif box. The 5′UTR or promoter sequences were entered in the input the sequences box. Advanced options were set *p* < 1.0E-4 and start search. Then frequencies of the background genes (DEGs in NX32-natural aging vs. NX32-0d) were also calculated.

The MEME was used to identified the enriched motif in 5′UTR, 3′UTR and promoter sequences. Briefly, the classical mode was selected for motif discovery. Sequences were uploaded into the primary sequence box. Motif width was set to 6–9 bp.

### Coexpression Regulatory Network

The network reconstruction was performed using the STRING application in Cytoscape (Shannon et al., 2003). Pearson’s correlation coefficient between AP2 transcription factor and targeted gene of > 0.7 (positive regulation) or < 0.7 (negative regulation) were used as a threshold and visualized using Cytoscape.

### RT-qPCR Analysis

For the mRNA expression analyses, total RNA was extracted from rice seeds using Trizol (Takara 9109, Japan). cDNA was synthesized by using Maxima H Minus First Strand cDNA Synthesis Kit (Thermo Fisher Scientific K1682, United States) following the manufacturer’s protocol. qPCR was performed using Bio-Rad CFX96 with SYBR Premix Ex Taq II (Innovagene SQ101-01, China). The primers used for the qPCR analysis are listed in Supplementary Table 4, and *OsACTIN* was used as an internal reference. The cDNAs were amplified following denaturation using 42-cycle programs (95°C, 15 s; 60°C, 20 s per cycle).

### Statistics

Significant differences in the data were analyzed by Student’s *t*-test or by multivariate comparison (one-way ANOVA) using SPSS (version 17.0) software. The significant differences of the changes during the aging between HL and LL were analyzed by multivariate comparison (two-way ANOVA).

## Results

### Classification of Rice Varieties by Seed Longevity After Aging

**FIGURE 1:**
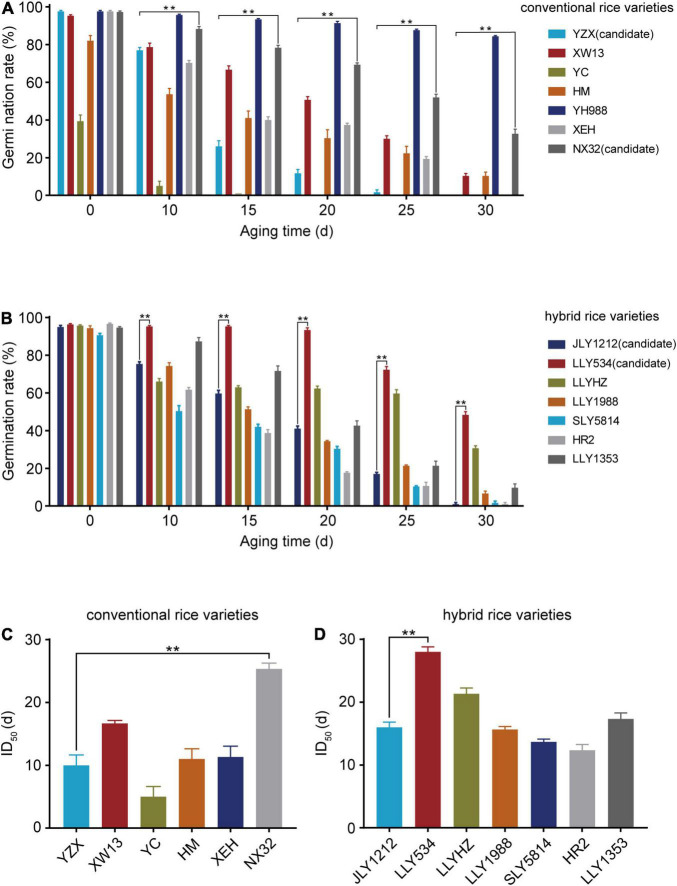
Germination rate and ID_50_ of conventional and hybrid rice seeds. **(A,B)** Germination rates of seven conventional **(A)** and seven hybrid rice varieties **(B)** after artificial aging treatment. The germination rates were recorded after the seeds germinated for 8 days. The experiments were repeated three times, and the error bars represent the SDs of three biological replicates (**P < 0.01, one-way ANOVA with Tukey’s test). **(C,D)** Half maximal inhibitory days (ID_50_) of six conventional **(C)** and seven hybrid varieties **(D)**. YH988 is not shown because it did not reach ID_50_ after 30 days artificial aging. Data are the means ± SDs based on three biological replicates (**P < 0.01, one-way ANOVA with Tukey’s test).

To obtain rice varieties with higher longevity (HL) and lower longevity (LL), we selected 14 rice varieties, including 7 conventional and 7 hybrid rice varieties (Supplementary Table 1). Then, we carried out artificial aging experiment and assessed seed longevity with a germination assay. Artificial aging led to a rapid decline in the germination rate of all rice varieties. Prior to aging, the germination rates of the YC and HM seeds were 39.3 and 82.1%, respectively, which were lower than those of other rice varieties (Figure 1A). The germination rates of YH998 and NX32 were significantly higher than those of the other varieties after aging, while the germination rate of YZX, XEH, and YC were relatively low (Figure 1A). In terms of the germination rate of hybrid rice varieties after aging, LLY534 and LLYHZ had higher germination rates, while JLY1212, SLY5814, and HR2 had lower germination rates (Figure 1B). Given that different rice varieties have different initial germination rate before aging, it is not accurate to use only the germination rate of seeds to evaluate seed longevity. The half inhibitory time (ID_50_) refers to the time that the seed germination rate is reduced to half of the non-aged germination rate. The larger the ID_50_ value is, the slower the seed germination rate decreases with aging, which reflects the higher longevity of the seeds. According to the results, YH998 did not reach half maximal inhibition even after 30 days of artificial aging, and NX32 had the highest ID_50_ (25.3 days). Since YH988 is a red rice that is rich in anthocyanins, which might have a role in anti-oxidation (Zhu, 2018), we chose NX32 as the HL seed variety. The ID_50_ values of YZX and YC were 9.8 and 5.2 days, respectively (Figure 1C). However, the initial germination rate of YC was much lower than that of the other varieties; therefore, we chose YZX as the LL seed variety among the conventional rice varieties. In hybrid rice, the ID_50_ value of LLY534 was the highest (27.2 days; Figure 1D), while the ID_50_ values of JLY1212, SLY5814, and HR2 were 16.0, 13.6, and 12.3 days, respectively (Figure 1D). In addition, the initial germination rate of SLY5814 was lower than that of the other varieties. Although the germination rate of HR2 were similar to JLY1212 after aging, the fatty acid content and eating quality of JLY1212 were worse after aging (data not shown), and JLY1212 having a wider planting area in China. Thus, LLY534 and JLY1212 were chosen for further research as the HL and LL seed varieties among the hybrid rice varieties. In summary, we obtained rice varieties with higher or lower longevity in both conventional rice and hybrid rice.

### Comparison of the Effects of Natural and Artificial Aging on Seed Longevity

**FIGURE 2:**
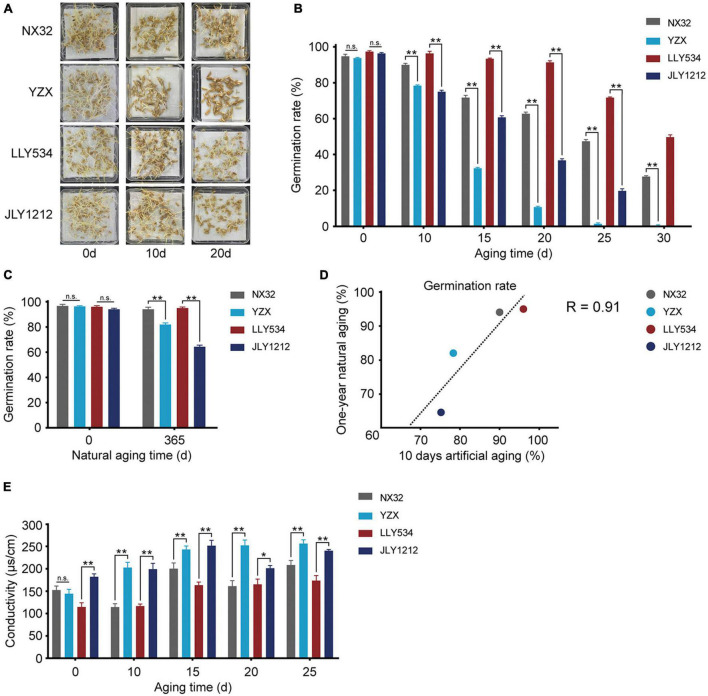
Germination rate and conductivity of seeds of the four rice varieties. **(A,B)** Germination rates of two conventional rice varieties (HL rice variety NX32 and LL rice variety YZX) and two hybrid rice varieties (HL rice variety LLY534 and LL rice JLY1212) under artificial aging conditions (0, 10, 15, 20, 25, and 30 days) (n.s., not significant; *P < 0.05, **P < 0.01, one-way ANOVA with Tukey’s test). **(C)** Germination rates of two conventional varieties (HL rice variety NX32 and LL rice variety YZX) and two hybrid varieties (HL rice variety LLY534 and LL rice variety JLY1212) under natural aging for 1 year (*P < 0.05, **P < 0.01). **(D)** Correlation analysis between natural aging (1 year) and artificial aging (10 d) of two conventional varieties (HL rice variety NX32 and LL rice variety YZX) and two hybrid varieties (HL rice variety LLY534 and LL rice variety JLY1212) (Pearson’s *R* = 0.91). **(E)** Seed conductivity of two conventional varieties (HL rice variety NX32 and LL rice variety YZX) and two hybrid varieties (HL rice variety LLY534 and LL rice variety JLY1212) under artificial aging conditions (0, 10, 15, 20, 25, and 30 d) (n.s., not significant; *P < 0.05, **P < 0.01, one-way ANOVA with Tukey’s test).

Aging is a natural process. A major drawback of natural aging is that it takes a long time, often approximately 1–2 years. Artificial aging, also known as the accelerated aging of seeds, costs a shorter time span, inducing the desired phenotypic changes in seeds (Hay et al., 2019). However, the effect of these two aging methods on the germination rate is still unclear in rice. To further compare the germination rates of NX32, YZX, LLY534, and JLY1212 under natural aging and artificial aging, we chose another batch of seeds for an additional experiment. NX32 had the highest seed longevity, and YZX had the lowest seed longevity (Figures 2A,B). Concerning the hybrid rice varieties, LLY534 had higher seed longevity, and its germination rate remained at approximately 48%, even after 30 days of artificial aging. Moreover, JLY1212 had lower seed longevity, and its seed vigor decreased rapidly compared with LLY534 after artificial aging (Figures 2A,B). In addition, the germination rates of NX32, YZX, LLY534, and JLY1212 after 1 year of natural aging were 94.1, 82.3, 95.6, and 64.3%, respectively (Figure 2C). We analyzed the correlation between the germination rate of seeds after 1 year of natural aging and 10 days of artificial aging and found that the correlation coefficient was high (Pearson’s *R* = 0.91; Figure 2D), suggesting that the effect of artificial aging treatment for 10 days was similar to the effect of 1 year of natural aging.

The cell membrane of rice seeds is often damaged during aging, and cytosolic solutes can flow into intercellular spaces, leading to an increase in the conductivity of the seed soaking solution (Panobianco et al., 2007). We then tested the electrical conductivity to evaluate the vigor of the seeds. Compared with NX32 rice seeds, those of YZX had a higher electrical conductivity increase after the artificial aging treatment (Figure 2E), and the electrical conductivity of JLY1212 was higher than that of LLY534 before and after aging (Figure 2E). These data indicated that electrical conductivity could be used as an indicator for evaluating seed longevity and that aging treatment might had a greater impact on the membrane integrity of LL rice varieties than that of HL rice varieties.

### Transcriptomic Analysis of Rice Varieties After Natural and Artificial Aging

**FIGURE 3:**
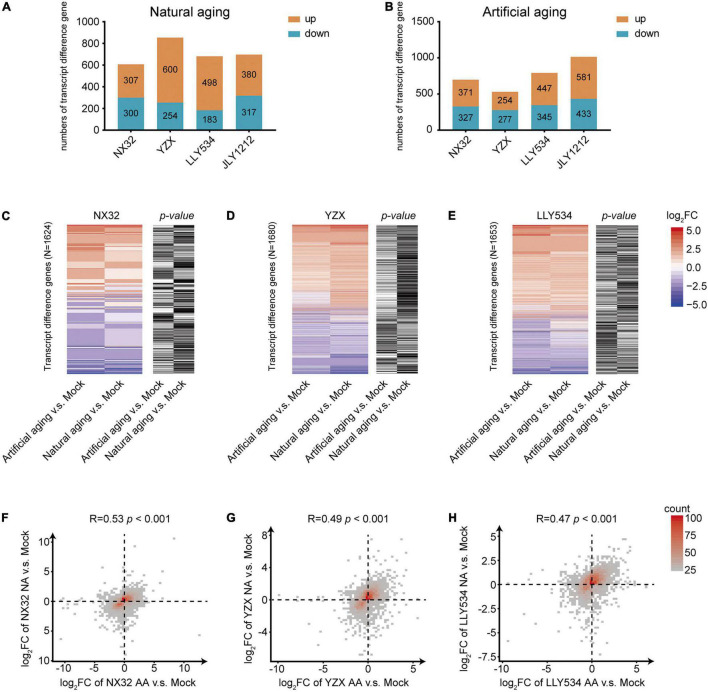
Comparison of gene expression between artificial and natural aging of HL and LL varieties. **(A,B)** Number of differentially expressed transcripts (| log_2_FC| > 1 and *p* < 0.05) in the four rice varieties after natural aging **(A)** and artificial aging **(B)** compared with mock conditions. **(C–E)** Heatmap of changes in gene expression in NX32 **(C)**, YZX **(D)** and LLY534 **(E)** after artificial aging and natural aging compared with mock treatment. Blue–red represents mRNA expression levels, where red represents higher mRNA expression levels, and blue represents lower mRNA expression levels. Black–white represents the *p-*value of the expression level of the transcripts. If the *p*-value is less than 0.05, it is black; otherwise, it is white. **(F–H)** Density figures showing the difference in NX32 **(F)**, YZX **(G)** and LLY534 **(H)** transcript changes (| log_2_FC| > 1 or *p* < 0.05) between artificial aging and natural aging. The x- and y-axes represent changes in the transcription level under artificial aging and natural aging, respectively. Spearman correlation was used for correlation analysis. NA represents natural aging, AA represents artificial aging.

The germination rate of rice seeds after artificial aging for 10 days was similar to that of seeds after natural aging for 1 year, suggesting that the effect of an appropriate artificial aging time could mimic the effect of natural aging for 1 year. To investigate the difference between natural and artificial aging at the transcriptional level, RNA-seq experiments were performed for rice seeds with 1-year natural aging or 10-days artificial aging. Regarding conventional rice varieties, NX32 treated with natural aging had 607 differentially expressed genes (DEGs) compared with the mock treatment (stored at −20°C for 1 year), of which 307 were upregulated and 300 were downregulated. In addition, 371 upregulated genes and 327 downregulated genes were identified in NX32 treated with 10-d artificial aging (Figures 3A,B and Supplementary Table 2; | log_2_FC| ≥ 1; *p*< 0.05). For the YZX rice variety treated with natural aging, there were 600 upregulated genes and 254 downregulated genes. For YZX treated with 10-d artificial aging, there were 254 upregulated genes and 277 downregulated genes compared with the mock treatment (Figures 3A,B and Supplementary Table 2). In hybrid seed varieties, 498 upregulated genes and 183 downregulated genes were identified in LLY534 treated with natural aging, and 447 upregulated genes and 345 downregulated genes were identified in LLY534 treated with 10-d artificial aging (Figures 3A,B and Supplementary Table 2). In addition, 380 upregulated genes and 317 downregulated genes were detected in JLY1212 treated with natural aging, and 581 upregulated genes and 433 downregulated genes were detected in JLY1212 treated with 10-d artificial aging (Figures 3A,B and Supplementary Table 2). Heatmap analysis indicated that most gene expression changes (*p* < 0.05 for artificial aging or natural aging) in NX32, YZX, and LLY534 in natural aging and artificial aging were correlated and changed in the same direction (Figures 3C–E). The overlapping gene changes (*p* < 0.05 for artificial aging or natural aging) between artificial aging and natural aging were in the same direction, and the values were moderately consistent in NX32 (*r* = 0.53, *p* < 0.05; Figure 3F), YZX (*r* = 0.49, *p* < 0.05; Figure 3G), and LLY534 (*r* = 0.47, *p* < 0.05; Figure 3H).

These results suggested that natural and artificial aging showed a similar effect on the transcription in rice seeds.

### Comparison of mRNA Expression Levels in Higher Longevity and Lower Longevity Rice Varieties After Natural or Artificial Aging

**FIGURE 4:**
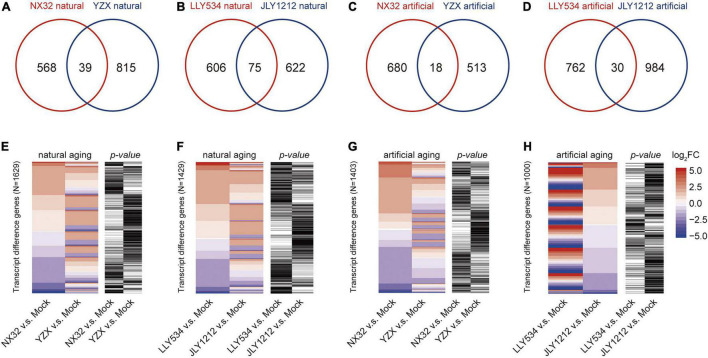
Comparison of significantly differentially expressed genes between four rice varieties with natural and artificial aging. **(A,B)** Venn diagram depicting the overlap of significant transcript differences (| log_2_FC| > 1 and *p* < 0.05) between NX32 and YZX **(A)** and between LLY534 and JLY1212 **(B)** under natural aging conditions. **(C,D)** Venn diagram depicting the overlap of significant transcript differences (| log_2_FC| > 1 and *p* < 0.05) between NX32 and YZX **(C)** and between LLY534 and JLY1212 **(D)** under artificial aging conditions. **(E,F)** Heatmap of differentially expressed genes in conventional varieties NX32 and YZX **(E)** and in hybrid varieties JLY1212 and LLY534 **(F)** when comparing natural aging with mock aging. Blue–red represents mRNA expression levels, and black–white represents the *p*-value of the expression level of the transcripts. If the *p*-value is less than 0.05, it is black; otherwise, it is white. **(G,H)** Heatmap of differentially expressed genes in conventional varieties NX32 and YZX **(G)** and in hybrid varieties JLY1212 and LLY534 **(H)** when comparing artificial aging with mock aging. Blue–red represents mRNA expression levels, and black–white represents the *p*-value of the expression level of the transcripts. If the *p*-value is less than 0.05, it is black; otherwise, it is white.

To test whether there is a difference in the expression of long-lived mRNAs between HL and LL varieties, we made a Venn diagram for the long-lived mRNA of these varieties under aging conditions. The results showed that the number of overlapping genes was relatively small across HL and LL varieties under both artificial and natural aging conditions. There were only 39 overlapping genes in NX32 and YZX under natural aging conditions (Figure 4A), 75 overlapping genes in LLY534 and JLY1212 under natural aging conditions (Figure 4B), 18 overlapping genes in NX32 and YZX under artificial aging conditions (Figure 4C) and 30 overlapping genes in LLY534 and JLY1212 under artificial aging conditions (Figure 4D). The overall expression trend of HL and LL rice varieties was determined based on the heatmap, which showed that some of the genes in NX32 and YZX were different under natural aging conditions (| log_2_FC| ≥ 1 for NX32 or YZX; *p* < 0.05 for NX32 or YZX; Figure 4E), while the same tendency was found in the comparison between LLY534 and JLY1212 with natural aging (Figure 4F) and NX32 and YZX with artificial aging (Figure 4G). In particular, the degree of mRNA changes is the most obvious between JLY1212 vs. Mock and LLY534 vs. Mock after artificial aging (Figure 4H), which is consistent with the lowest germination rate of JLY1212 after aging. These results indicated that there are certain differences in the transcription levels between HL and LL rice varieties after aging.

### Comparison of Gene Ontology Terms in Higher Longevity and Lower Longevity Rice Varieties After Natural Aging

**FIGURE 5:**
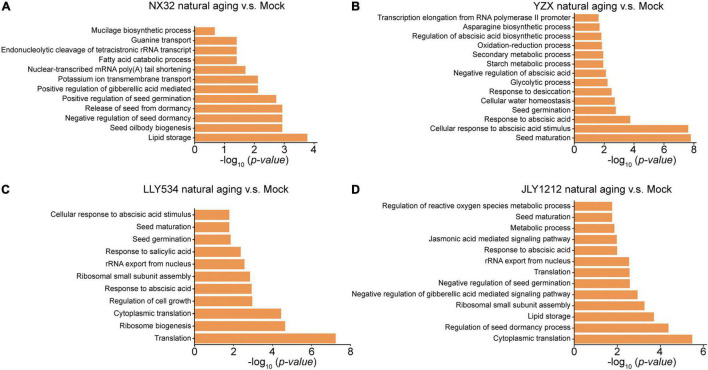
Gene Ontology (GO) enrichment of genes with significant expression changes after natural aging treatment compared with mock treatment in four rice varieties. **(A–D)** GO terms of NX32 **(A)**, YZX **(B)**, LLY534 **(C)** and JLY1212 **(D)** after natural aging. The y-axis represents the functional subcategory, and the x-axis represents the value of the −log_10_(*p*-value), which indicates the significance level.

To further analyze the difference in biological pathways between HL and LL rice varieties, we compared the Gene Ontology (GO) terms for DEGs in HL and LL rice varieties under different aging conditions. In conventional rice varieties, GO analysis showed that DEGs in NX32 after natural aging were involved in lipid storage, seed oil body biogenesis, negative regulation of the seed dormancy process, release of seeds from dormancy and positive regulation of seed germination. In addition, GO terms related to stress hormones were also enriched, such as response to positive regulation of the gibberellic acid-mediated signaling pathway (Figure 5A). Moreover, DEGs in YZX after natural aging functioned in seed maturation, cellular response to abscisic acid stimulus, response to abscisic acid, seed germination, cellular water homeostasis and response to desiccation (Figure 5B), especially the enrichment of seed maturation and cellular response to abscisic acid stimulus. The DEGs of the two conventional rice varieties after aging were mostly related to the processes of seed vigor, dormancy, and germination. Besides, there were also certain differences, the most enriched GO terms of the YZX variety were seed maturation and cellular response to abscisic acid stimulus (Figure 5B), while lipid storage and seed oil body biogenesis was enriched in NX32 (Figure 5A). In hybrid rice varieties, GO term analysis showed that DEGs in LLY534 after natural aging for 1 year were involved in translation, ribosome biogenesis, response to abscisic acid and seed germination (Figure 5C). However, DEGs in JLY1212 after natural aging for 1 year functioned in cytoplasmic translation, regulation of seed dormancy process, lipid storage and negative regulation of gibberellic acid mediated signaling pathway (Figure 5D). Similarly, the main signaling pathways enriched in LLY534 and JLY1212 also showed certain differences, which also coincided with the greater difference between the DEGs in the HL and LL varieties. In summary, the main enriched biological pathways of HL and LL rice varieties after 1 year of aging have certain differences, which may be one of the reasons for the difference in seed longevity.

### Analysis of the Specific Long-Lived RNAs for Seed Longevity

**FIGURE 6:**
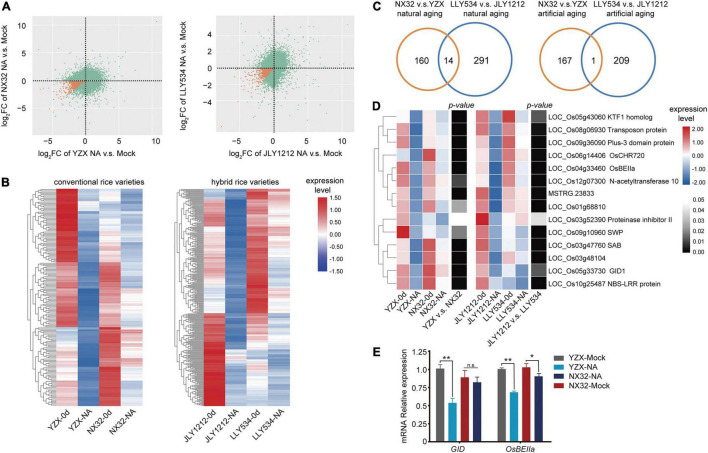
Heatmap analysis of long-lived mRNAs in HL and LL varieties. **(A)** Figure showing the log_2_FC in NX32, YZX, LLY534, and JLY1212 when comparing natural aging with mock treatment (*p* < 0.05). The orange dots represent the specific long-lived mRNA. NA represent natural aging. **(B)** Heatmap of specific long-lived mRNAs that degrade significantly slower in HL varieties NX32 and LLY534 than in LL varieties YZX and JLY1212 when comparing naturing aging with mock treatment (*p* < 0.05). **(C)** Venn diagram depicting the overlap of specific long-lived mRNAs that degrade significantly slower in HL varieties than in LL varieties (*p* < 0.05) between NX32 vs. YZX and LLY534 vs. JLY1212 under natural and artificial aging conditions. **(D)** Heatmap of overlapping specific long-lived mRNAs that degrade significantly slower in HL varieties than in LL varieties (*p* < 0.05) between conventional rice varieties (NX32 and YZX) and hybrid rice varieties (LLY534 and JLY1212) under natural aging conditions. NA represents natural aging. Black–white represents the *p*-value of the changes during the aging between HL and LL. **(E)** qRT-PCR analysis of *GID1* and *OsBEIIa* expression in NX32 and YZX with or without natural aging. *OsACTIN* was used as the internal control. The data are presented as the mean ± SD (*n* = 3) (n.s., not significant; *P < 0.05, **P < 0.01, one-way ANOVA with Tukey’s test).

Previously, it has been reported that the stability of embryonic RNAs required for germination is related to seed longevity (Saighani et al., 2021). These long-lived mRNAs play important roles in the process of protein synthesis during the initial phase of seed germination. Because most transcripts were degraded during aging, we selected transcripts that were down regulated in HL varieties but had a slower degradation rate than that of LL varieties [log_2_FC HL < 0 and log_2_FC LL < 0 and (log_2_FC HL-log_2_FC LL) > 0] as the long-lived mRNAs (*p* < 0.05 for HL or LL) (Figures 6A,B). By two-way ANOVA, we screened out these special long-lived mRNAs that degrade significantly slower (*p* < 0.05 for the changes during the aging between HL and LL) in HL varieties than in LL varieties. In conventional rice, 174 long-lived mRNAs were identified in NX32 v.s. YZX after natural aging (Figure 6B and Supplementary Table 3). In hybrid rice, 305 long-lived mRNAs were identified in LLY534 v.s. JLY1212 after natural aging (Figure 6B and Supplementary Table 3). The degradation rate of these long-lived mRNAs is slower after aging in HL varieties, and the degradation rate of these long-lived mRNAs is more rapid after aging in LL varieties. To identify more reliable long-lived mRNAs that participate in the regulation of seed vigor in both conventional rice and hybrid rice, we used Venn analysis to identify the overlapping genes in the NX32 v.s. YZX and LLY534 v.s. JLY1212 comparisons (Supplementary Figures 1A,B) and 14 overlapping genes were identified under natural aging conditions (Figures 6C,D, Supplementary Figure 2, and Supplementary Table 5). Of them, GID1 (LOC_Os05g33730) is gibberellin receptor, indicating that the GA pathway may be related to seed vitality. In addition, LOC_Os04g33460, a starch branching enzyme IIa (OsBEIIa), was also identified. Further, we analyzed the expression of *GID1* and *OsBEIIa* in HL and LL varieties seeds with or without natural aging using qPCR. The results were consistent with the RNA-seq data (Figure 6E), indicating the reliability of the RNA-seq data. In addition, in conventional rice, 168 long-lived mRNAs were identified in NX32 v.s. YZX after artificial aging (Supplementary Figure 1B and Supplementary Table 3). In hybrid rice, 210 long-lived mRNAs were identified in LLY534 v.s. JLY1212 after artificial aging (Supplementary Figure 1B and Supplementary Table 3). One overlapping genes (LOC_Os02g10180) was identified in the comparison of NX32 vs. YZX and LLY534 v.s. JLY1212 after artificial aging. And only a few genes overlapped between artificial aging and natural aging in NX32 v.s. YZX or LLY534 v.s. JLY1212 comparisons (Supplementary Figure 1A). Since the number of overlapping long-lived mRNA identified under naturing aging is more abundant, we used them in the follow-up analysis.

It has been suggested that the mRNA stored in the mature seed is related to the ribonucleic acid protein complex, indicating that they are translated during seed germination. It has identified a conserved motif, GAAGAAGAA, which is significantly enriched at the 5′UTR and present at low levels in general seed ribosome-associated transcripts (Bueso et al., 2013). However, we did not find this motif enriched in the 14 overlapping long-lived mRNA. We analyzed whether these 14 overlapping long-lived mRNAs have similar sequence features in the promoter, 5′UTR or 3′UTR. It showed that three repeats of the sequence GGCGGCGGC was enriched in the promoter (*p* = 1.3E-3, percentage = 83.3%, background percentage = 51.3%; Supplementary Figure 3 and Supplementary Table 6). In addition, this *cis*-element was recognized by the AP2/EREBP transcription factors family (Castro-Mondragon et al., 2022). The AP2/DREBP transcription factor family plays a crucial role in seed development, seed storage metabolism and seed longevity (Okamuro et al., 1997; Cernac and Benning, 2004; Pereira Lima et al., 2017). The mRNA expression levels of AP2/EREBP transcription factor members in naturing aging were analyzed and their DEGs data were used to build a possible transcriptional regulation pathway on rice longevity regulation mediated by AP2/EREBPs (Supplementary Figures 4A–D). In summary, we identified 14 specific long-lived mRNAs that might be important to seed longevity. The gibberellin receptor gene GID1 suggested that is GA pathway may be involved in seed vigor.

## Discussion

Long-lived mRNAs are very important for seed vigor, and their degradation has been detected alongside viability loss in seeds (Fleming et al., 2018). It has been reported that RNA is more vulnerable to oxidation by ROS than DNA due to its single-strandedness (Kong and Lin, 2010), the oxidation of mRNA is not random but selective (Bazin et al., 2011), and damaged mRNA cannot be translated, which will lead to a loss of seed longevity. Dry seeds often serve as the final point of seed development or the initial step in the seed germination series during transcriptomic analysis (Bai et al., 2017; Pereira Lima et al., 2017). It lacks of study on the changes in the transcriptome during seed storage and the identification of specific mRNAs associated with longevity. In this study, we firstly identified 2 HL rice varieties and 2 LL rice varieties by screening 14 rice varieties (7 conventional and 7 hybrid varieties) with artificial aging, and analyzed the effect of artificial and natural aging on transcriptional events. We found that most gene expression changes in the HL and LL varieties under natural and artificial aging were correlated, indicating that artificial and natural aging have similar effects on transcription events. In addition, our results suggested that the degradation of some transcripts occurred specifically during aging, which is consistent with the highly selective nature of RNA oxidation, as some mRNAs are more susceptible to oxidative damage or targeted oxidation (Shan et al., 2003; Chang et al., 2008; Bazin et al., 2011). However, this result differs from a previous result showing that transcripts were degraded non-specifically (Fleming et al., 2018). Previous studies have shown that artificial aging (CDT method) and natural aging have similar effects at the level of protein abundance changes (Rajjou et al., 2008). However, the similarity between CDT and artificial aging during seed longevity is controversial (Nguyen et al., 2012; Buijs et al., 2018, 2020), as different QTLs are involved in seed longevity depending on the seed aging protocol used (Nagel et al., 2011, 2015; Arif et al., 2017). Different aging methods have different main effects on seeds, which might be one of the reasons for this controversy. Changsha has a subtropical monsoon climate, the air is humid all year, and the temperature in summer is higher than that during the rest of the year; thus, natural aging occurs under conditions of high humidity, which may be one of the reasons why the transcriptomes are similar under both natural aging and artificial aging (high temperature and high humidity). Therefore, the artificial aging method in this research could mimic the natural aging method in high-humidity areas. Moreover, we found RNA is much more prone to oxidative modifications than DNA, even during anhydrobiosis, which would lead to the abundance of oxidized transcripts changing during the after-ripening dry period (Bazin et al., 2011). During rice seed storage, the embryo still has a certain level of activity due to the high humidity in the environment, which leads to the expression of genes related to DNA repair or RNA processing. These might the reason that some mRNAs increased after aging.

During seed maturation, long-lived mRNAs required for the initial stage of germination are synthesized and then stored in the seeds until they are required. Long-lived mRNA will be degraded in the process of seed storage, which inevitably affects the reduction of seed vigor. To identify the long-lived mRNAs that play an important role in seed vigor, we mainly compared the DEGs in HL and LL varieties after natural aging. The heatmap of HL and LL varieties showed that there were certain differences in the expression of some genes in these varieties after aging, and this difference was more obvious in JLY1212 under artificial aging, which might indicate that JLY1212 was more intolerant to storage. At the same time, there were some differences in the GO term enrichment of the HL and LL varieties after natural aging, especially regarding seed maturation, seed dormancy and lipid storage. The degree of enrichment of signaling pathways, such as response to ABA, response to salicylic acid and regulation of GA, also differs between HL and LL varieties; these pathways are associated with seed vigor (Zhao et al., 2021). In addition, we identified 14 special long-lived mRNAs, and their expression levels were significantly different in HL and LL varieties after aging. A motif involved in the initial process of seed germination was enriched in the promoter of GID1. It has been reported that gibberellin can promote seed germination (Yamaguchi and Kamiya, 2001), and gibberellin has an inhibitory effect on seed deterioration (Bueso et al., 2016); seeds treated with GA are more tolerant to aging. The GA 20-oxidase (AtGA20ox) and GA 3-oxidase (AtGA3ox) catalyzed successive steps in the synthesis of bioactive GAs, which had highly lower transcript levels in AtGID1-overexpressing plants than in wild-type plants. Overexpression of AtGID1 increased the sensitivity of *Arabidopsis* to GA (Ju et al., 2018), suggesting a potential role of GID1 in seed longevity. In addition, three AtGID1 receptors have partially specialized functions in seed germination in *Arabidopsis*, AtGID1c play positive regulator of seed germination, whereas AtGID1b negatively regulate germination in dormant seeds in the dark (Ge and Steber, 2018). There are several putative GA receptor genes in rice (Miao et al., 2019), therefore, different GID1 homologous genes may play different roles in rice seed longevity. The search for genes in the GA signaling network may be important for the study of seed longevity (Bueso et al., 2014).

ABA is the other major phytohormones in seed development and seed vigor regulations. It reported that OsHIPL1 protein may modulate endogenous ABA levels and altering OsABIs expression and interacts directly with OsPIP1;1 to affect seed vigor in rice (He et al., 2022), we analyzed the expression of OsHIPL1 and OsPIP1;1 in the HL and LL varieties (Supplementary Figures 5A,B), unfortunately, their expression levels have no difference between two varieties, suggesting the differences between the reverse genetic method and the forward genetic method (e.g., transcriptomic analysis). We also identified several long-lived mRNAs with unknown functions, which might be the missed or omitted regulators in rice longevity. The identification of specific long-lived mRNAs in seeds would help to design genetic approaches for using mutants of these mRNAs to understand the mechanisms of genes involved in seed storability regulation in the future. Further work will validate the role of the characterized genes in seed longevity and explore the mechanism by which they are regulated by transcription factor AP2/EREBP.

## Data Availability Statement

The datasets presented in this study can be found in online repositories. The names of the repository/repositories and accession number(s) can be found below: GSA database, https://ngdc.cncb.ac.cn/bioproject/browse/PRJCA006248.

## Author Contributions

LW conceived the project and designed the research. BW, YT, LJ, and WH performed the research. FY and QL contributed to new reagents and analytical tools. BW and SW analyzed the RNA-seq data. BW and LW wrote the manuscript. All authors reviewed and approved the manuscript for publication.

## Conflict of Interest

The authors declare that the research was conducted in the absence of any commercial or financial relationships that could be construed as a potential conflict of interest.

## Publisher’s Note

All claims expressed in this article are solely those of the authors and do not necessarily represent those of their affiliated organizations, or those of the publisher, the editors and the reviewers. Any product that may be evaluated in this article, or claim that may be made by its manufacturer, is not guaranteed or endorsed by the publisher.

## Footnotes

1
http://geneontology.org/

2
http://rice.uga.edu/
