# Supplementary material for: Transcriptome-Wide Characterization of Seed Aging in Rice: Identification of Specific Long-Lived mRNAs for Seed Longevity
Source: Front Plant Sci. 2022 May 16;13:857390. doi: 10.3389/fpls.2022.857390 (PMC9149411; doi:10.3389/fpls.2022.857390)
Supplement: Supplementary file 7 [file Data_Sheet_1.pdf]

Supporting information

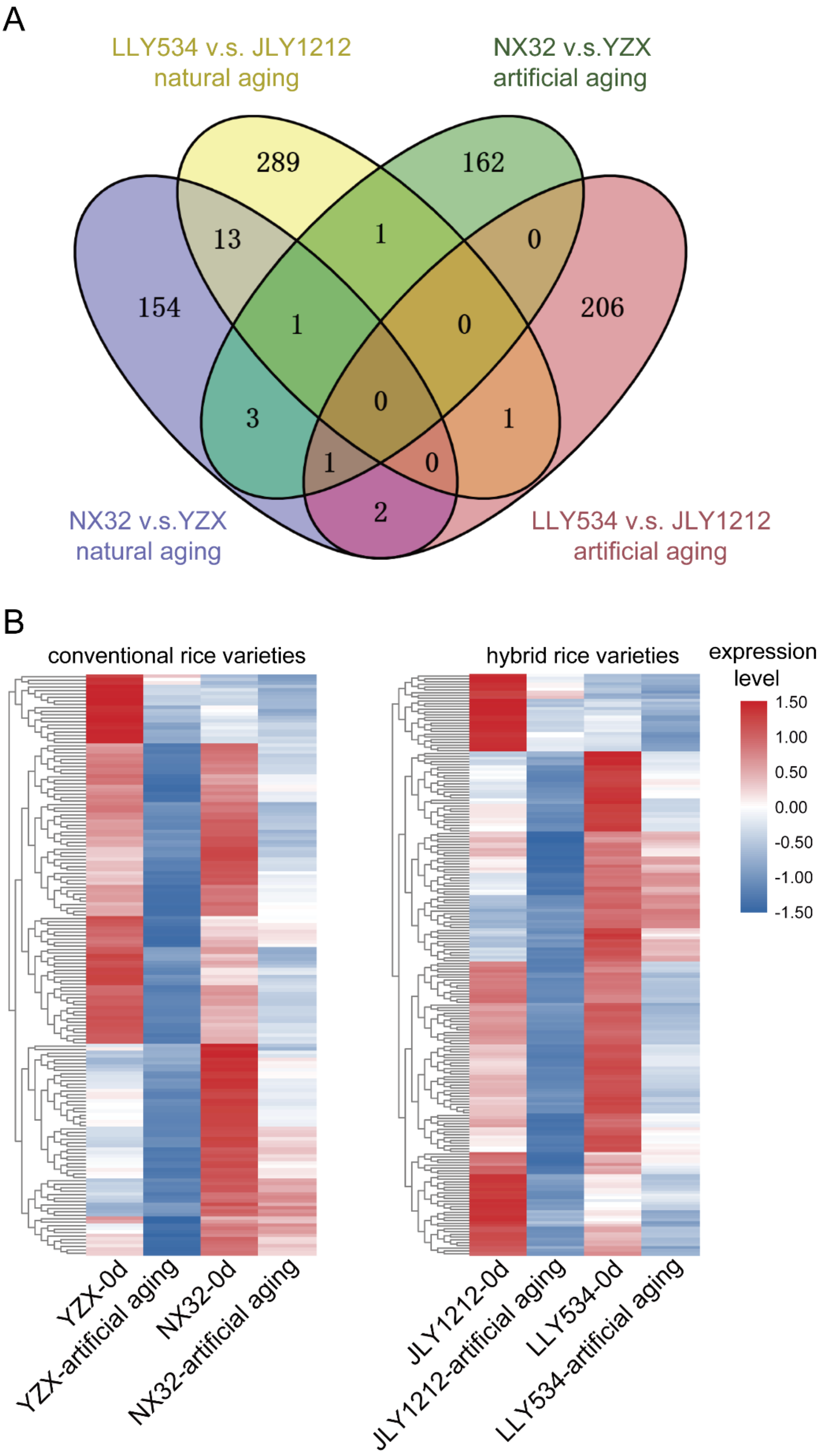

Fig. S1. Heatmap analysis of long-lived mRNA after artificial aging. (A) Venn diagram depicting the overlap of specific long-lived mRNAs that degrade significantly slower in HL varieties than in LL varieties (*p-value* < 0.05) between natural aging conditions and artificial aging conditions. **(B)** Heatmap of specific long-lived mRNAs that degrade significantly slower in HL varieties NX32 and LLY534 than in LL varieties YZX and JLY1212 when comparing artificial aging with mock treatment (*p-value* < 0.05).

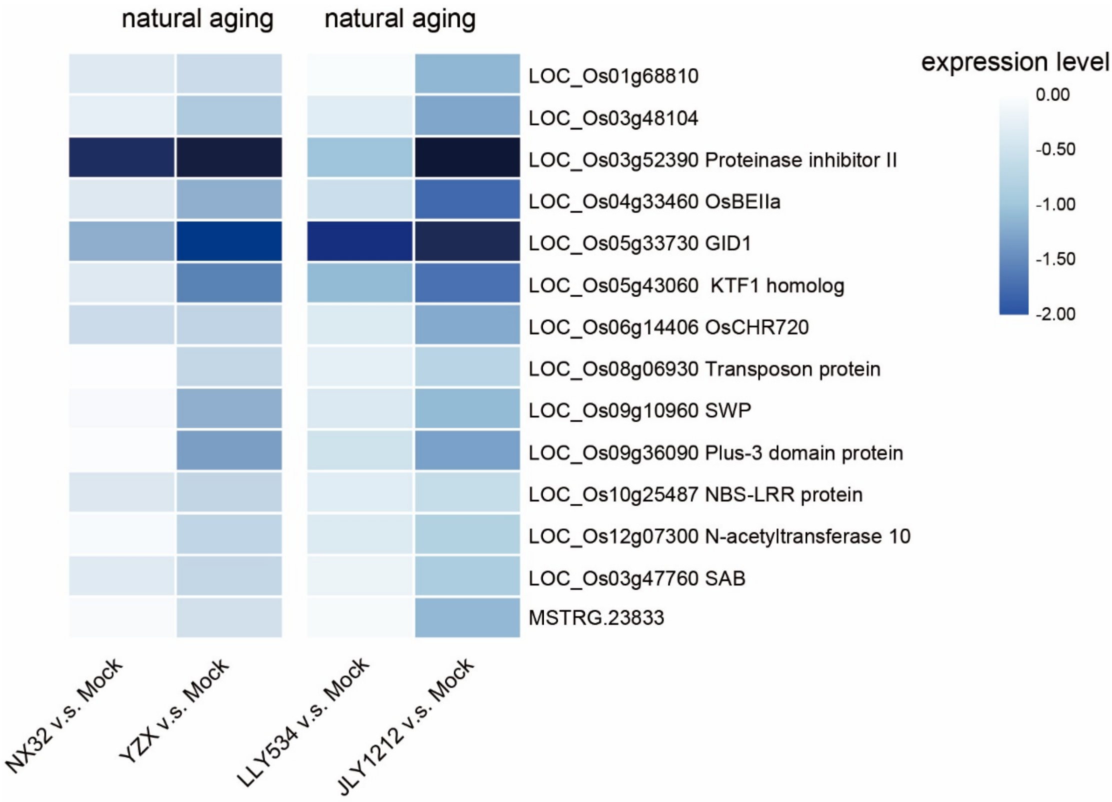

Fig. S2. Heatmap analysis of long-lived mRNAs in HL and LL varieties.

Heatmap of overlapping specific long-lived mRNAs that degrade significantly slower in HL varieties than in LL varieties (*p-value* < 0.05) between conventional rice varieties (NX32 and YZX) and hybrid rice varieties (LLY534 and JLY1212) under natural aging conditions.

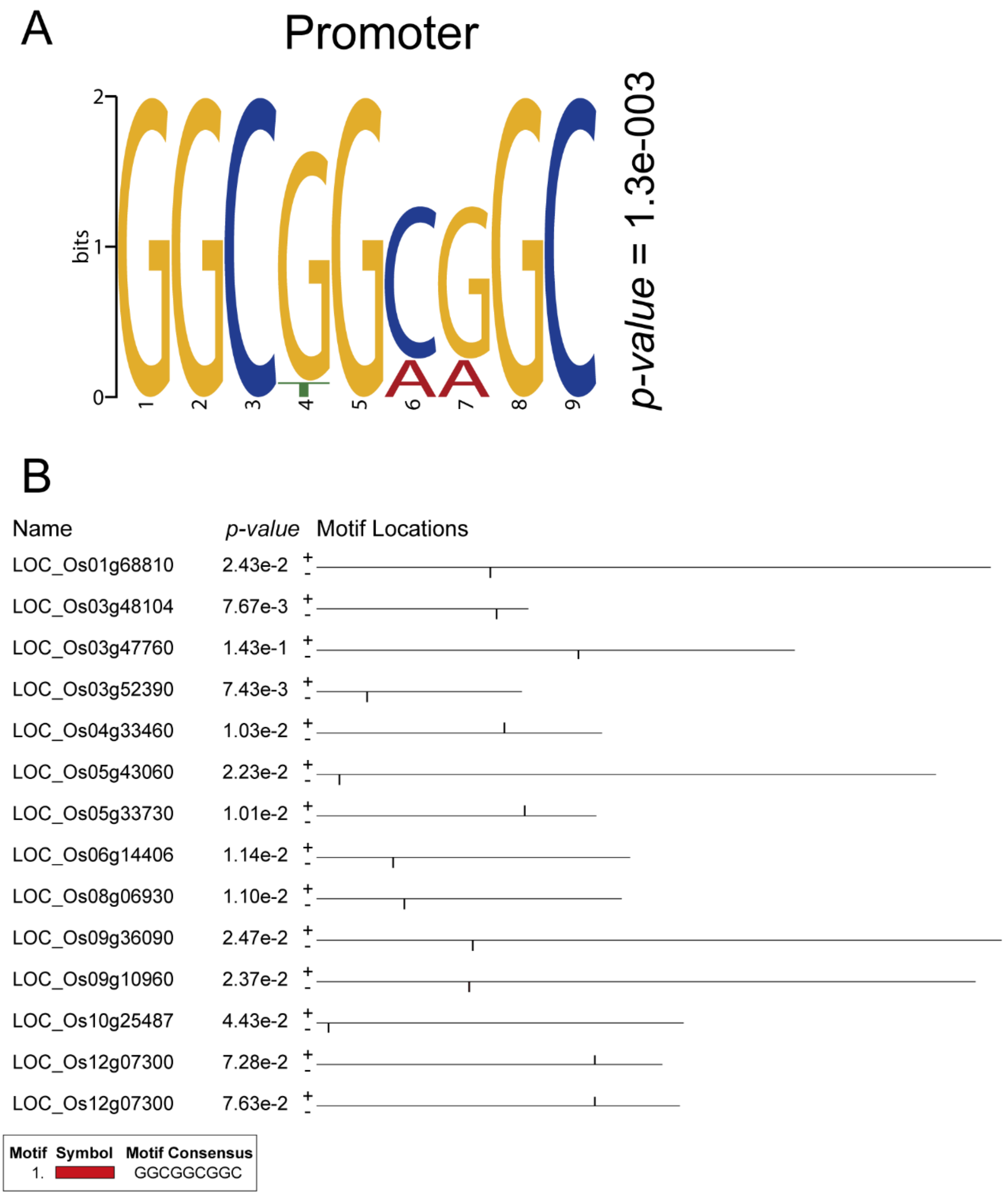

Fig. S3. Identified similar sequence features of promoter in the specific long-lived mRNA. **(A)** Motif analysis of enriched motif by MEME. The E-value is the enrichment *p-value* multiplied by the number of candidate motifs tested. The motif was identified as one of the top enriched motifs. (**B**) Schematic diagram of the positions of putative motifs in the 5' UTR or promoter sequences. 14 genes that overlapped between NX32 and JLY1212 under natural aging conditions (Fig.6C) were used to the analysis of enriched motif.

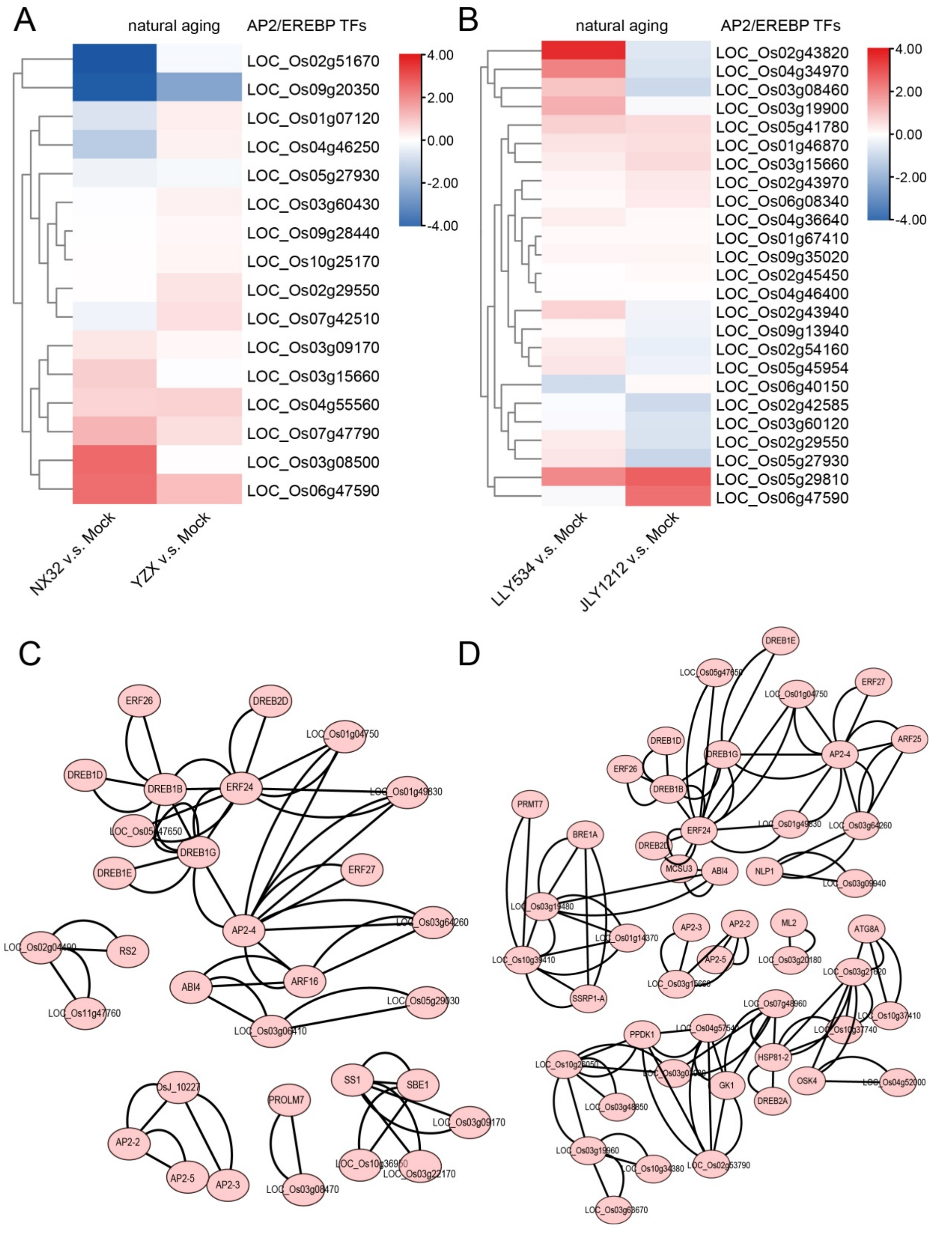

Fig. S4. Heatmap analysis of AP2/EREBP transcription factor in HL and LL varieties. **(A-B)** Heatmap of AP2/EREBP transcription factor family genes in HL varieties (NX32 and LLY534) and in LL varieties (YZX and JLY1212) when comparing natural aging with mock treatment (*p-value* < 0.05 for HL or LL). **(C-D)** The identification of gene-regulated long-lived mRNA from the coexpression network of TFs and targeted long-lived mRNAs in conventional and hybrid rice,

respectively.

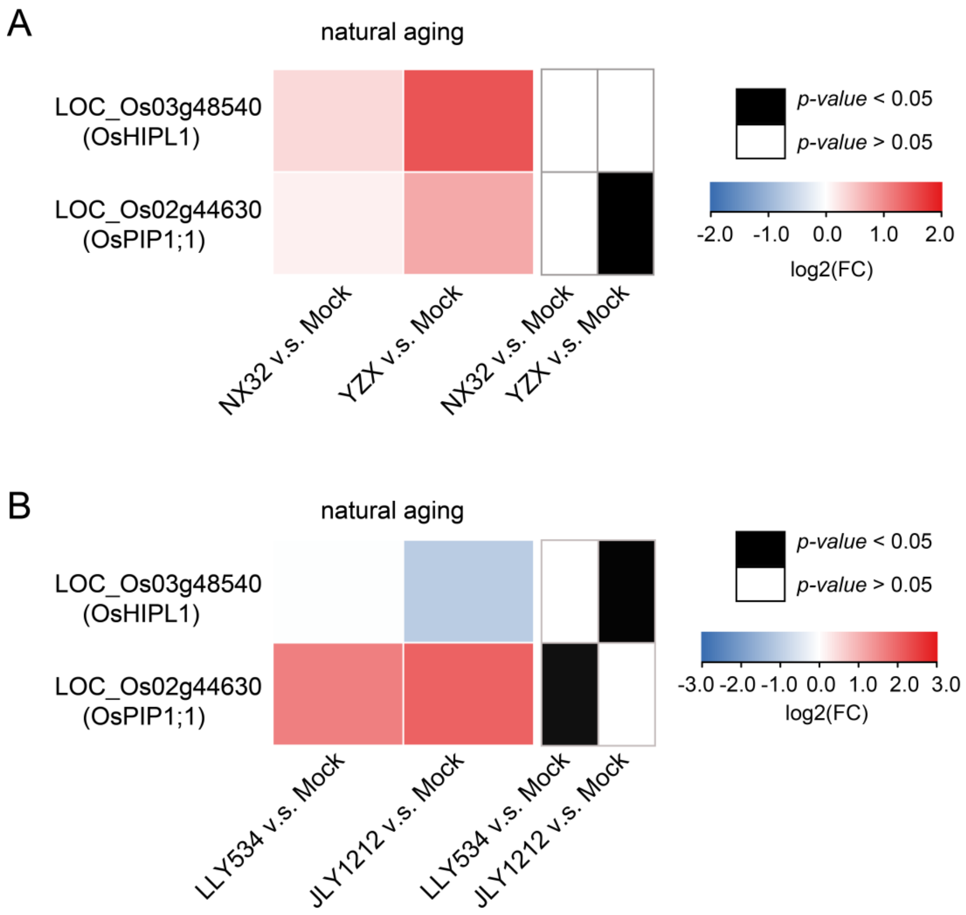

Fig. S5. mRNA expression of OsHIPL1 and OsPIP1;1 in HL varieties (NX32 and LLY534) and in LL varieties (YZX and JLY1212) when comparing naturing aging with mock treatment. (A-B) Heatmap of OsHIPL1 and OsPIP1;1 between conventional rice varieties (NX32 and YZX) and hybrid rice varieties (LLY534 and JLY1212) under natural aging conditions. Black–white represents the *p-value* of the changes during the aging between HL and LL.
